# Deep-brain magnetic stimulation promotes adult hippocampal neurogenesis and alleviates stress-related behaviors in mouse models for neuropsychiatric disorders

**DOI:** 10.1186/1756-6606-7-11

**Published:** 2014-02-11

**Authors:** Yan Zhang, Rong-Rong Mao, Zhi-Fang Chen, Meng Tian, Da-Li Tong, Zheng-Run Gao, Min Huang, Xiao Li, Xiu Xu, Wen-Hao Zhou, Cheng-Yu Li, Jiang Wang, Lin Xu, Zilong Qiu

**Affiliations:** 1 Institute of Neuroscience, Shanghai Institutes for Biological Sciences, Chinese Academy of Sciences, Shanghai, China; 2 Key Laboratory of Animal Models and Human Disease Mechanisms, Laboratory of Learning and Memory, Kunming Institute of Zoology, Chinese Academy of Sciences, Kunming, Yunnan, China; 3 School of Electrical and Automation Engineering, Tianjin University, Tianjin, China; 4 Departments of Neonatology, Children’s Hospital of Fudan University, Shanghai, China; 5 Department of Child Healthcare, Children’s Hospital of Fudan University, Shanghai, China

**Keywords:** Deep-brain magnetic stimulation, Adult hippocampal neurogenesis, Long-term potentiation, Depression, MeCP2, Rett syndrome

## Abstract

**Background:**

Repetitive Transcranial Magnetic Stimulation (rTMS)/ Deep-brain Magnetic Stimulation (DMS) is an effective therapy for various neuropsychiatric disorders including major depression disorder. The molecular and cellular mechanisms underlying the impacts of rTMS/DMS on the brain are not yet fully understood.

**Results:**

Here we studied the effects of deep-brain magnetic stimulation to brain on the molecular and cellular level. We examined the adult hippocampal neurogenesis and hippocampal synaptic plasticity of rodent under stress conditions with deep-brain magnetic stimulation treatment. We found that DMS promotes adult hippocampal neurogenesis significantly and facilitates the development of adult new-born neurons. Remarkably, DMS exerts anti-depression effects in the learned helplessness mouse model and rescues hippocampal long-term plasticity impaired by restraint stress in rats. Moreover, DMS alleviates the stress response in a mouse model for Rett syndrome and prolongs the life span of these animals dramatically.

**Conclusions:**

Deep-brain magnetic stimulation greatly facilitates adult hippocampal neurogenesis and maturation, also alleviates depression and stress-related responses in animal models.

## Background

Transcranial magnetic stimulation (TMS) is a non-invasive approach of brain stimulation, which utilizes an insulated coil placed over the scalp and induces neural activity within the brain [1-3]. TMS is commonly applied in single, paired or repetitive trains. Repetitive TMS (rTMS) has been proven to modulate motor skills and cognitive function in healthy subjects and exhibits therapeutic effects for patients with neurological and psychiatric disorders [4,5]. Daily prefrontal TMS was approved by Food and Drug Administration (FDA) in 2008 for the treatment of patients with major depressive disorder. Recently, deep-brain magnetic stimulation (DMS) with a modified rTMS protocol has been developed and demonstrated to be effective for Parkinson’s disease and neuropsychiatric disorders, including depression [6,7]. Despite the indubitable contribution of rTMS/DMS to cognitive and motor functions of nervous system, the molecular and cellular mechanisms underlying the anti-depression effects of rTMS/DMS remain largely unknown. The prevailing hypothesis is that rTMS may stimulate neural activity in certain brain regions by modulating the balance between excitatory and inhibitory neurons [8-12].

Depression is a leading cause of psychiatric disability worldwide. During the past decade, the adult neurogenesis hypothesis of depression has been widely accepted, which postulated that the decline in adult hippocampal neurogenesis contributes to the pathophysiology of depression, while the biogenesis of adult new-born neurons in the dentate gyrus (DG) of hippocampus is required for the beneficial effects of antidepressant treatment [13-15]. In non-human primates, stress led to a decrease in adult hippocampal neurogenesis, which was rescued by antidepressant treatment [16]. Chronic rTMS treatment exhibited robust anti-depression effects in animal models and might enhance adult neurogenesis under chronic stress [17,18].

Here we show that DMS increases the proliferation of hippocampal neural progenitor cells in the adult brain, promotes the dendritic complexity of new-born neurons and enhances the neuronal activity in hippocampus, indicated by the up-regulation of activity-dependent genes. Moreover, administration of DMS not only alleviates the depression and anxiety-associated behaviors of a mouse model for Rett syndrome, but also strikingly prolongs the lifespan of these animals.

## Results and discussion

### DMS promotes adult hippocampal neurogenesis

**Figure 1:**
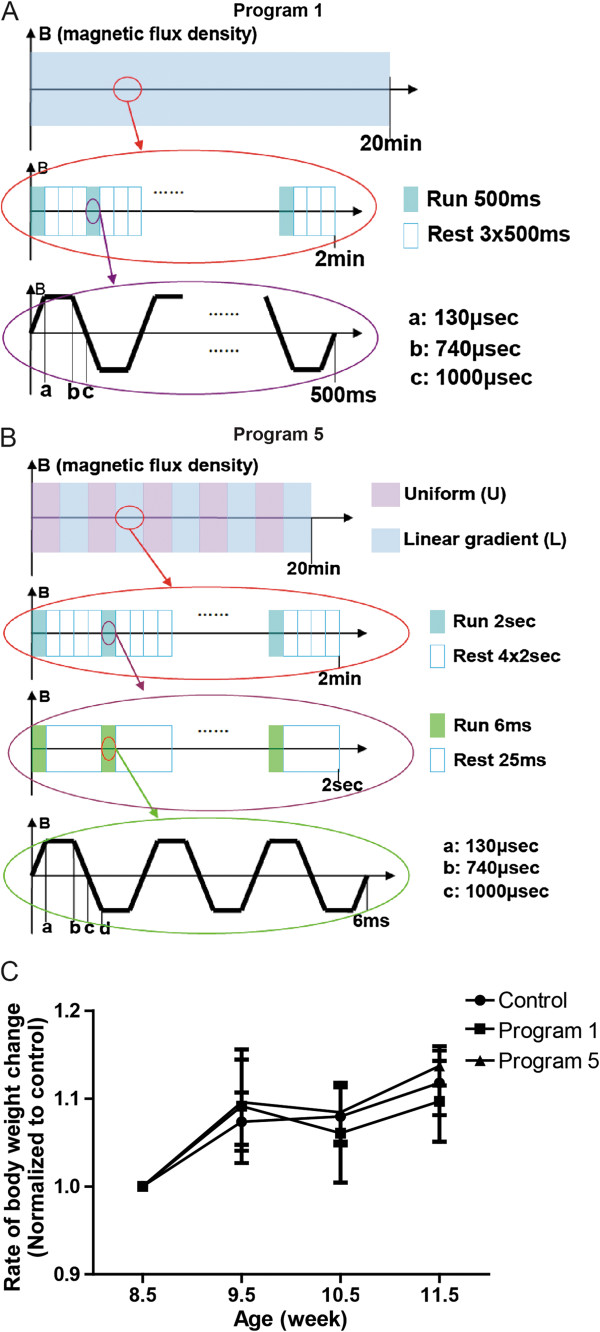
**Schematic illustration of the new DMS paradigm. (A)** Illustration of Uniform and Linear magnetic fields generated by this equipment. Composition of Program 1 **(A)** and Program 5 **(B)**. **(C)** Body weight changes of mice under control, Program1, and Program5 treatment daily for 3 weeks. (n = 6 for each condition).

To examine the effects of DMS on adult hippocampal neurogenesis in wild-type animals, we applied the DMS protocol to 6 weeks old wild-type mice 20 minutes daily. The schematic illustration of the DMS device is shown in (Additional file 1: Figure S1A). Two magnetic stimulating coils are symmetrically placed on the both sides of the mouse cage. The stimulus device can be discharged through two coils to produce a time-varying pulsed magnetic field. The first stimulating procedure (Program1) is shown in Figure 1A. In this strategy, the magnetic flux density is linear gradient. The magnetic pulse is bipolar, as shown in Additional file 1: Figure S1C,E. The second stimulating paradigm (Program 5) is shown in Figure 1B. Different with Program1, the magnetic flux density of Program 5 is uniform alternating linear gradient, both magnetic fields are illustrated in Additional file 1: Figure S1B-E. First, we examined whether DMS stimulation would have global effects on the general health of experimental animals. We measured the body weights of mice receiving either Program 1 (P1) or Program 5 (P5) treatment daily for 3 weeks and found that P1 or P5 treatment didn’t affect the body weight changes comparing to naïve mice (Figure 1C).

**Figure 2:**
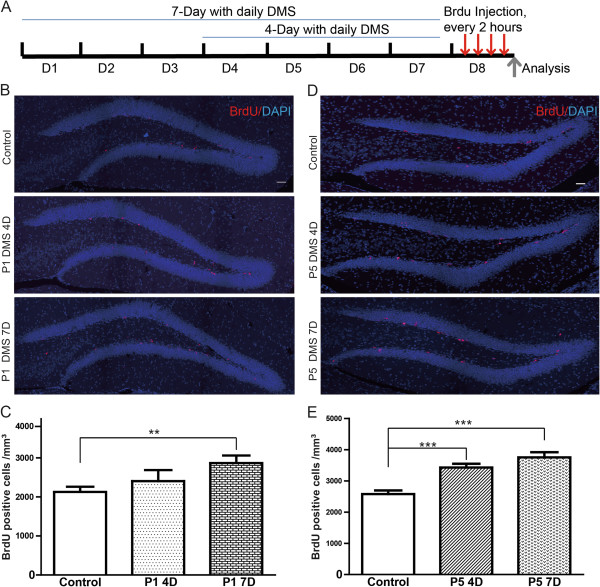
**DMS promotes adult hippocampal neurogenesis in rodents. (A)** Outline of DMS and BrdU injection experiments. **(B)** Hippocampal DG BrdU immunostaining on mice with continuous DMS with Program 1 treatment. Red: BrdU; Blue: DAPI staining. **(C)** Quantification of **(B)**. Values represent mean (±) SEM (n = 3 animals for each condition; **: P < 0.005, two-tailed student’s t-test). **(D)** Hippocampal DG BrdU immunostaining on mice with continuous DMS with Program 5 treatment. Red: BrdU; Blue: DAPI staining. **(E)** Quantification of **(D)**. Values represent mean (±) SEM (n = 3 animals for each condition; ***: P < 0.0005, two-tailed student’s t-test). Scale bar = 50 μm.

To investigate the role of DMS in the proliferation of neural stem cells, we applied a short-term bromodeoxyuridine (BrdU) labeling strategy to monitor postnatal hippocampal neurogenesis in the mice subjected to DMS with P1 or P5, respectively. For the thymidine analog incorporation assay, BrdU was administered into adult mice 4 times with 2-hour interval by intraperitoneal injection after 4 or 7 days treatment of DMS (Figure 2A). The treated animals were sacrificed 2 hours after the last time of BrdU injection. The staining of BrdU labels the new-born neurons in the hippocampus. The results showed a dramatic increase in the number of active proliferating neural progenitor cells in the subgranular zone of DG after administration of DMS with P1 for 7 days rather than 4 days (Figure 2B,C). Intriguingly, stimulation with P5 significantly elevated the production of new-born neurons in the DG as early as 4 days after treatment, suggesting that different DMS paradigms may generate various biological effects for adult neurogenesis (Figure 2D,E).

**Figure 3:**
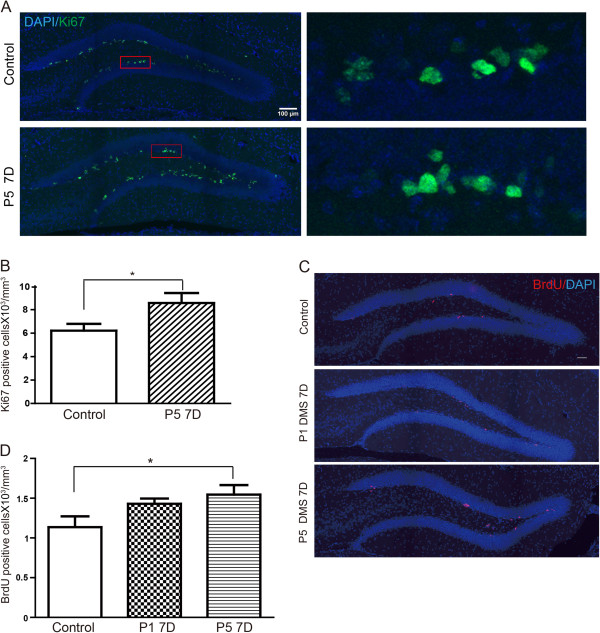
**DMS promotes neural progenitors proliferation in rodents. (A)** Hippocampal DG Ki67 immunostaining on mice with continuous DMS with Program 5 treatment for 7 days. Blue: DAPI; Green: Ki67 staining. Right panel: Amplification of boxed area of left panel. Scale bar = 100 μm. **(B)** Quantification of **(A)**. **(C)** Hippocampal DG BrdU immunostaining on 9 month old mice with continuous DMS with Program 1 or 5 treatment. **(D)** Quantification of **(C)**. Values represent mean (±) SEM (Animal amounts: Control n = 4, P1 n = 3, P5 n = 4; *: P < 0.05, two-tailed student’s t-test). Scale bar = 50 μm.

To further confirm whether DMS treatment enhances proliferation of neural progenitors, we further performed immunostaining using antibody against Ki67, a cell proliferating marker on these samples. We found that the number of Ki67- positive cells was significantly increased in DG area after P5 treatment for 7 days, indicating that DMS treatment promotes neural progenitor proliferation (Figure 3A,B). To validate that the BrdU-labeled cells are proliferating progenitors, we performed co-immunostaining for both BrdU and Ki67 and found 100% of colocalization between the two markers. (Additional file 2: Figure S2A-D). Furthermore, to reveal the cellular identity of BrdU-positive cell we performed immunostaining using antibodies against more neural progenitor markers including radial glial-like cell (RGL) marker glial fibrillary acidic protein (GFAP), immature neuron marker doublecortin (DCX), and intermediate progenitor (IPC) marker T-box brain 2 (Tbr2). We found that cells labeled by short-term BrdU pulse are during actively proliferating process and show 63% (control) versus 67% (DMS) co-localization with Tbr2, 21% (control) versus 16% (DMS) with GFAP, and 14% (control) versus 15% (DMS) with DCX (Additional file 2: Figure S2E-H and Additional file 3: Figure S3A-H). Taken together, our results indicated that DMS promotes not only the self- renewal of RGLs but also their differentiation into IPCs and late neuroblasts.

Next, we examined the effect of DMS treatment on adult hippocampal neurogenesis by in aged animals, in which the rate of adult neurogenesis remains in a much lower level than adult mice. The observation that aging has a negative effect on the proliferation of neural stem cells [19] has called up efforts to boost neurogenesis in senescent animals, which will have beneficial effects for age-related cognitive decline. To study whether DMS improves the reduced neurogenesis in senescent mice, we applied daily DMS in 9-month old mice for 7 days and examined the neurogenesis by BrdU incorporation assay. Our results revealed that the population of proliferating neural progenitors was significantly enhanced after DMS treatment in P5, while DMS with P1 had a slight increase but not statistically significant effects on hippocampal neurogenesis (Figure 3C,D). Thus, we demonstrated that DMS with patterned stimulation protocols promoted the proliferation of hippocampal neural progenitor cells in both adult and senescent animals.

### DMS facilitates the development of new-born neurons

Adult hippocampal neurogenesis largely recapitulates the process of neural development in embryonic stages. After birth, new-born neurons in the DG migrate into the granule cells layer, extend dendrites toward the molecular layer, project axons through the hilus toward the CA3 and integrate into the existing circuitry [20,21].

**Figure 4:**
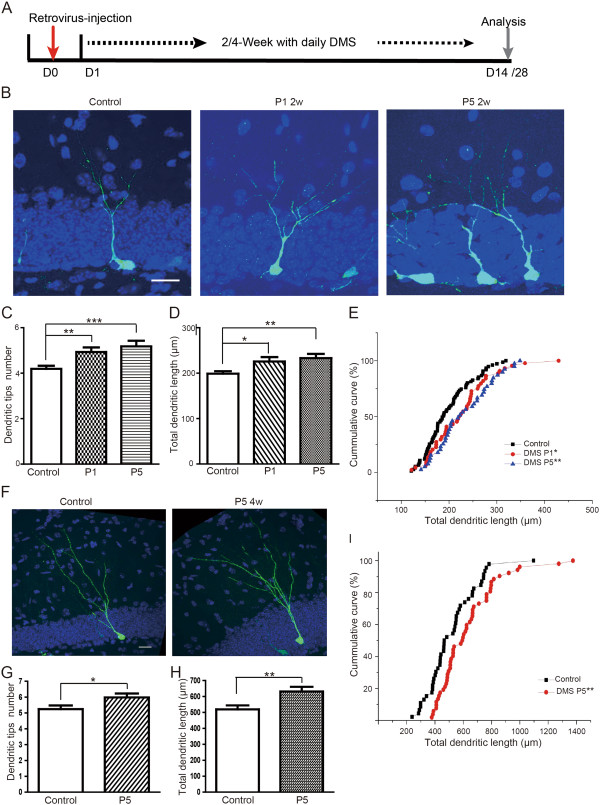
**DMS facilitates development of DG new-born neurons. (A)** Outline of DMS and retroviral-based new-born neuron label experiments. **(B)** GFP immunostaining with retroviral labeled new-born neurons after DMS treatment for 2 weeks. Green: GFP; Blue: DAPI staining. **(C,D)** Total dendritic length and tip numbers measured from **(B)** Values represent mean (±) SEM (n = 4 animals for each group; **: P < 0.005, ***: P < 0.0005, two-tailed student’s t-test). **(E)** Accumulative curve of dendritic length of neurons undergoing control, P1 and P5 treatment. (**F)** GFP immunostaining with retroviral labeled new-born neurons after DMS treatment for 4 weeks. Green: GFP; Blue: DAPI staining. **(G,H)** Total dendritic length and tip numbers measured from **(F)** Values represent mean (±) SEM (Animal amounts, control:n = 4, P5:n = 3; **: P < 0.005, *: P < 0.05, two-tailed student’s t-test). Scale bar = 25 μm.

To address the effects of DMS on the maturation of new-born neurons, we labeled these neurons by stereotactic injection of retrovirus into the DG of young mice, followed by the 2-week daily treatment of DMS with distinct programs (Figure 4A) [22]. Retroviral infection of proliferating neural progenitor cells with the introduction of green fluorescent protein (GFP) allows the sparse labeling of new-born neurons and the elaborate observation of their morphological complexity. The morphology of GFP labeled new-born neurons was examined with confocal microscopy at 2 weeks post-injection (Figure 4B). The results showed that there was a significant increase in both the number of the tips and total dendritic length after DMS treatment with either P1 or P5, when compared to the control GFP labeled neurons (Figure 4C-E). We further applied DMS with Program 5 up to 4 weeks to mice which received retrovirus injection ahead of stimulation and analyzed the dendritic morphogenesis of new-born neurons (Figure 4F). Consistently, the results revealed that DMS robustly facilitated the dendritic growth and branching of neurons (Figure 4G-I).

In order to examine whether DMS may affect the development of mature neurons, we performed Golgi staining on the brain sections from control and DMS-treated animals (Additional file 4: Figure S4A,B). We measured the spine density of DG granule neurons after DMS for 2 weeks and found that DMS has no effects on spine density of fully mature neurons (Additional file 4: Figure S4C,D).

Collectively, these evidences demonstrated that DMS treatment not only enhances the proliferation of adult neural progenitor cells in DG, but also facilitates the development and maturation of new-born neurons, suggesting that new-born neurons induced by DMS are able to incorporate into hippocampal neural circuitry and contribute to neuronal plasticity of the central nervous system.

### DMS stimulates gene expression in hippocampus *in vivo*

**Figure 5:**
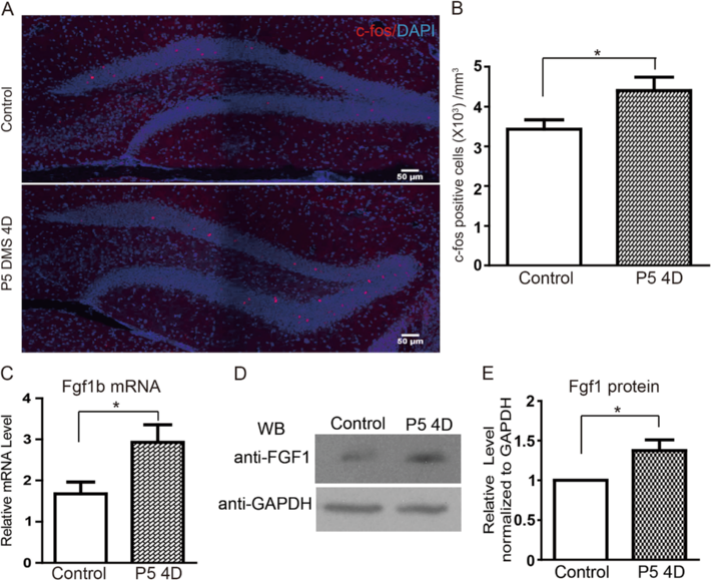
**DMS stimulates gene expression in hippocampus. (A)** Immunohistochemistry for c-fos expression in hippocampal DG region after DMS with P5 treatment for 4 days. **(B)** Quantitation for c-fos positive cells in per 1 mm^3^. Values represent mean (±) SEM (n = 4 for each conditions; *: P < 0.05, student’s t-test). **(C)** mRNA level of *fgf1b* gene in hippocampus after DMS with P5 for 4 days. Values represent mean (±) SEM (n = 4 for each conditions; *: P < 0.05, student’s t-test). **(D)** Protein level of Fgf1b after DMS P5 treatment for 4 days. Hippocampal tissue samples were collected from control and animals received P5 daily treatment for 4 days. Samples were homogenized and analyzed by SDS-PAGE using antibodies indicated. **(E)** Quantification of **(D)**.

To further explore the consequences at the molecular level caused by DMS treatment, we performed immunostaining and quantitative PCR to examine the change of gene expression in the hippocampus *in vivo*. Neural activity induces the modification of synaptic proteins, promotes local protein synthesis within dendrites and activates gene transcription in neurons. Therefore, we compared the expression level of several activity-dependent genes in the hippocampus between control groups and DMS-treated groups. The immediate early genes including *c-fos, egr1* and *arc* are commonly used as markers for measuring neuronal activity in the brain. We first examined the expression of immediate early gene *c-fos,* in the DG after daily DMS with P5 paradigm for 4 days by immunohistochemistry. The results of immunostaining showed that the population of c-fos-positive neurons was significantly increased in DMS-treated animals (Figure 5A,B). Additionally, the Fgf1b gene is a brain-specific expressing gene encoding an important neurogenic niche factor in the adult hippocampus, and is induced by electroconvulsive stimulation [23]. We collected the hippocampal samples from animals with or without DMS treatment, further examined the mRNA levels of *fgf1b* in the DG by quantitative PCR. We found that the expression level of *fgf1b* was remarkably elevated after DMS (Figure 5C). Furthermore, we examined the protein level of Fgf1 in hippocampus with DMS treatment for 4 days. Consistently, we found that DMS treatment for 4 days significantly lead to an increase of protein level of Fgf1 in hippocampus (Figure 5D,E). These results suggest that DMS stimulation increases neural activity in the hippocampus, induces the expression of activity-dependent gene and leads to the release of neurogenic niche factors, which thereby promotes neurogenesis and plasticity in the adult brain.

### DMS exerts anti-depression effects on rodent model

**Figure 6:**
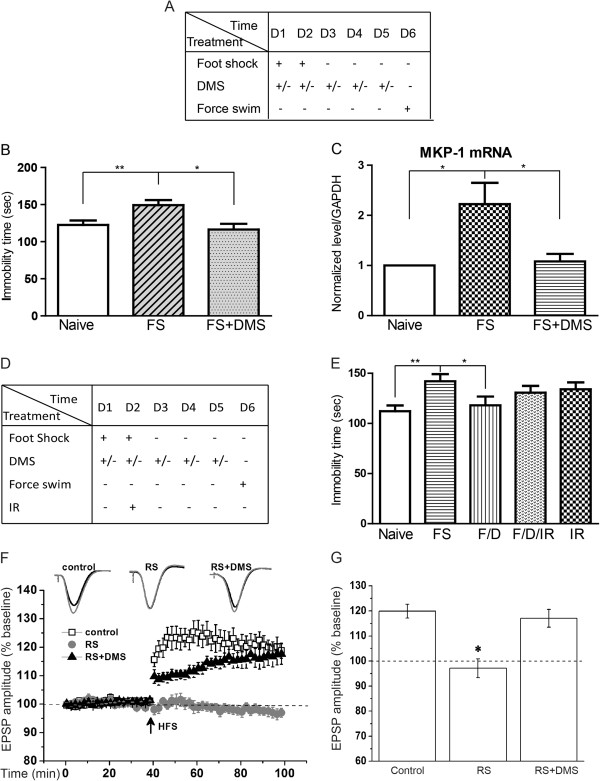
**DMS rescues stress-induced phenotypes and synaptic plasticity in rodent models. (A)** Outline of foot-shock-induced depressive behavioral paradigm and DMS treatment experiments. **(B)** Behavioral effects of learned helplessness mouse model with DMS treatment. Values represent mean (±) SEM (Animal amount: control n = 15, Foot-shock n = 19, Foot-shock with DMS n = 15; **: P < 0.005, two-tailed student’s t-test. **(C)** Hippocampal Mkp-1 mRNA level in a learned helplessness mouse model with DMS treatment. Values represent mean (±) SEM (n = 12 animals for each group; *: P < 0.05, two-tailed student’s t-test). **(D)** Outline of foot-shock-induced depressive behavioral paradigm, DMS treatment, and gamma irradiation experiments. **(E)** Behavioral effects of learned helplessness mouse model with DMS and IR treatment. Values represent mean (±) SEM (n = 12-16 for each condition; **: P < 0.005, *: P < 0.05, two-tailed student’s t-test). **(F)** DMS treatment restored hippocampal LTP impaired by the chronic restraint stress. Compared with control group (n = 5, 119.92 ± 2.78% of baseline ), the LTP induction was significantly impaired in Restraint Stress (RS) group (n = 5, 97.10 ± 1.67% of baseline) and restored in RS + DMS group (n = 12, 117.06 ± 3.54% of baseline); **(G)** Summary of LTP induction in control, RS and RS + DMS group (F(2,19) = 8.64, *p = 0.002, compared with control group). Statistical comparisons in electrophysiological studies were made by the least significance difference test of one-way ANOVA. The significance level was set as *:p < 0.05.

It is postulated that the defects in adult hippocampus neurogenesis is strongly associated with major depressive disorder. Exercise may alleviate depression symptoms via enhancing neurogenesis in animals [24]. The rTMS has been an effective therapeutic tool for the treatment of several neuropsychiatric disorders in human patients, as well as rats with chronic unpredicted mild stress (CUMS) [25]. The evidence that application of DMS to wild type animals increases adult neurogenesis prompted us to investigate whether the newly-developed DMS protocol has anti-depressive effects in the rodent model. We applied learned helpless animal paradigm to model some aspects of depression in rodents [26]. Following 2 days of unpredicted electric foot-shocks, we successfully induce the depression phenotype in mice assessed by forced swimming test, in which mice with foot-shocks appeared to have increased immobility time compared to naïve animals (Figure 6A,B, Additional file 5: Figure S5A-E). After treating mice with continuous 5-day DMS treatment with P5, we found that the immobility time of depressed mice induced by foot-shock was fully rescued to normal level by the DMS treatment (Figure 6B).

The pathophysiology of major depressive disorder has been characterized by alternations of molecular markers. Whole-genome expression profiling revealed that mitogen-activated protein kinase phosphatase-1 (MKP-1) was dysregulated in the hippocampal tissues from patients with depression, suggesting that MKP-1 serves as a molecular marker for depression [27]. Consistently, the expression level of MKP-1 was significantly up-regulated in the hippocampus of mouse model with depression phenotypes (Figure 6C). Importantly, the dys-regulated expression of MKP-1 in depressed mice was completely restored by the DMS treatment (Figure 6C).

Next we investigated whether increased adult hippocampal neurogenesis induced by DMS plays a critical role in alleviation of depressive behavior by elimination of neurogenesis using gamma irradiation (IR). After applying IR after foot shock, we found that IR abolished the effect of DMS on alleviation of depressive behaviors (Figure 6D,E). Meantime, we found that IR itself also leads to a dramatic decrease of hippocampal adult neurogenesis (Additional file 6: Figure S6A-C).

Furthermore, mood disorder induced by acute stress impairs long-term potentiation (LTP) in the neural circuitry of hippocampus [28-31]. The effects on synaptic plasticity occur following a plethora of stressors including administration of shock, exposure to a predator or a novel environment [32]. We used chronic restraint stress paradigm to induce depression in rats and recorded LTP in hippocampus by *in vivo* electrophysiological recording. Indeed, LTP was found to be impaired in rats undergoing restraint stress (Figure 6F). Notably, DMS treatment for 7 days largely rescued the deficit LTP induction in depressed rats (Figure 6F,G). These behavioral, molecular and electrophysiological evidences strongly support that short-term DMS treatment is effective for alleviating depression-associated phenotypes in animal models.

### DMS alleviates anxiety-associated phenotypes in a mouse model for Rett syndrome

Finally we would like to further examine whether DMS could alleviate the anxiety-related behaviors in other disease models. Rett syndrome is a severe neural developmental disorder, primarily caused by *loss-of-function* mutations of gene *MECP2* (Methyl-CpG-binding protein 2) [33-35]. The mouse carrying a truncated form of MeCP2 protein (*mecp2*^*308/y*^) mimics severe phenotypes of Rett syndrome patients, including elevated anxiety and stress responses [36]. Thus we asked whether DMS could help to relieve the anxiety-associated phenotypes in mice carrying *mecp2*^*308/y*^ mutation.

**Figure 7:**
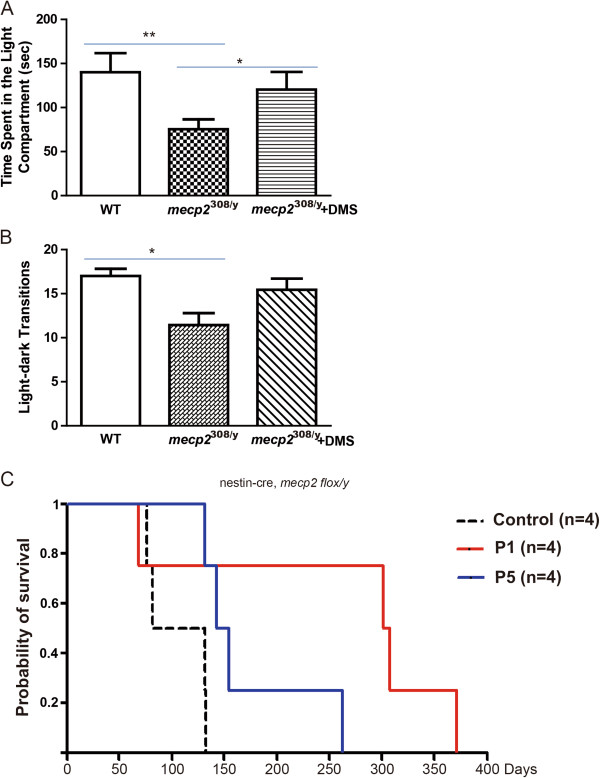
**DMS alleviates anxiety-associated defects and extend life span in mouse model for Rett syndrome. (A)** Time spent in light compartment during light–dark transition tests. Values represent mean (±) SEM (Animal amounts, wt n = 8, *mecp2*^*308/y*^ n = 9, *mecp2*^*308/y*^ + DMS n = 9; **: P < 0.005, *: P < 0.05, two-tailed student’s t-test). **(B)** Times of light–dark transitions during test. Values represent mean (±) SEM (*: P < 0.05, two-tailed student’s t-test). **(C)** Kaplan-Meier survive curve of nestin-cre, *mecp2*^*flox/y*^ mice with control, DMS with P1 and P5 treatments, respectively. (n = 4 for each condition).

We first examine stress responses in *mecp2*^*308/y*^ mouse and its wild type littermates, using light–dark transition paradigm. We found that mice carrying *mecp2*^*308/y*^ mutant appeared to spend much less time in light compartment and thus to have increased anxiety levels (Figure 7A,B). Interestingly, chronic daily DMS treatment for 5 months restored elevated anxiety levels of *mecp2*^*308/y*^ mice to normal level (Figure 7A,B). Thus, DMS treatment appears to have significant effects to regulate the anxiety levels of animals for disease models.

Lastly, we asked whether DMS treatment would make positive impacts to the general health of the mouse model of Rett syndrome. Lack of MeCP2 in mouse would lead to significant shortage of lifespan [37,38]. We acquired nervous system specific *mecp2* knockout mice by crossing mice carrying neuronal specific Cre transgene *nestin-cre* with *mecp2* floxed allele- *mecp2*^*flox/y*^. We found that loss of MeCP2 in the nervous system would lead to substantial shortage of lifespan down to around 130 days or so (Figure 7C). Surprisingly, mice with *nestin-cre*, *mecp2*^*flox/y*^ received daily DMS with either P1 or P5 appeared to have enormously extended lifespan (Figure 7C), indicating that long-term treatment of DMS indeed exerts positive effects on the general health to mouse model of Rett syndrome.

### Discussion

The growing interest in noninvasive brain stimulation generated by TMS led to its widespread application to treat various neurological and psychiatric disorders including major depression and Parkinson’s disease. The NeuroStar TMS Therapy system (Neuronetics, Inc., Malvern, PA, USA) received FDA clearance for the treatment of adult patients with intractable depression in 2008. It is assumed that magnetic stimulation makes use of electromagnetic principles to alter neural activity non-invasively, and induces focal as well as network effects in the brain. However, how rTMS changes the cellular behavior and functional connectivity remains enigmatic.

Here we developed a novel rTMS paradigm, referred as DMS, to help the learned helpless animals recuperate from stress and depression. The short-term application of DMS to depressed mice not only improved their stress-related behavior but also amazingly reversed the pathophysiology of major depression, indicated by the alteration of molecular marker and neuronal plasticity. As the neurogenic hypothesis of depression gains momentum over the last decade, more and more evidence confirmed that the waning and waxing of neurogenesis in the hippocampus are important causal factors in the precipitation of and recovery from depression, respectively. Our study provided several lines of evidences to prove that DMS treatment increases adult neurogenesis and new-born neuron maturation in vivo. First, short-term DMS administration rapidly induces the proliferation of adult neural progenitors in the subgranular zone of DG. The enhanced neurogenesis by DMS treatment occurs in both adult and senescent animals. Second, the application of DMS to adult animals promotes the dendritic development new-born granule neurons, suggesting the elevated incorporation of new-born neurons into existing hippocampal circuitry might be achieved by magnetic stimulation. Finally, DMS treatment is shown to up-regulate the neural activity, which is beneficial to increase the production and release of neurogenic niche factors such as FGF1b. Thus, our results support the notion that DMS is an efficient therapeutic treatment for major depression disorders and the antidepressant effects of DMS may rely on the elevation of hippocampal adult neurogenesis and the modulation of hippocampal synaptic plasticity.

Neural activity and experience, presumably acting on this local niche, regulate multiple stages of adult neurogenesis, from neural progenitor proliferation to newborn neuron maturation, synaptic integration and survival. Our study shows a new non-invasive way to enhance adult hippocampal neurogenesis probably by changing the network activity of hippocampus and inducing a beneficial neurogenic niche in the subgranular zone. Repetitive electrical stimulation in adult hippocampal slices induces NMDA receptor-dependent LTP and high frequency stimulation results in persistent increase in synaptic strength [39]. Studies using theta-burst stimulation protocols provide solid evidence linking human rTMS with LTP-like plasticity [40,41]. The DMS protocol we applied exerts high frequency oscillated stimulation in the brain of animal models and rescues the deficient LTP impaired by restraint stress in rats. On the other hand, LTP induction in the anesthetized rats increases neurogenesis in the dentate gyrus and FGFR activation by the neural cell adhesion molecule promotes neural progenitor proliferation by enhancing LTP [42,43]. Therefore, the study suggests that DMS administration may promote adult neurogenesis by inducing high frequency theta-burst stimulation and LTP in the hippocampus. Further study will be performed to explore the alteration of field potential by DMS treatment. Moreover, the finding that DMS alleviates stress response and prolongs the life expectancy of mouse model for Rett syndrome is particularly intriguing. Many trials have been carried out to examine whether drug delivery or genetic manipulation help to alleviate the impaired behavior and severe pathophysiology of Rett syndrome mouse model [44-46]. Although gene delivery of *Mecp2* seems to completely rescue various symptoms in animal models of disease, the efficacy and safety issues of gene delivery remain controversial. Here our results suggest a modified DMS paradigm which may relieve the stress-related symptoms, as well as improve the life quality of Rett syndrome patients. Further study of how DMS treatment may contribute the neural plasticity of the central nervous system under disorder conditions are very critical for deepen our understanding of therapeutic efforts towards the cure of neurodevelopmental disorders and neuropsychiatric disorders. In order to develop easier and more effective therapy for patients with neurodevelopmental disorders and neuropsychiatric disorders, we need to better understand how DMS treatment contributes to the neural plasticity of central nervous system under pathological conditions.

## Conclusion

Taken together, we report that a new DMS paradigm rapidly induces adult hippocampal neurogenesis and promotes development of DG new-born neurons. Our study provides a new non-invasive way to enhance adult hippocampal neurogenesis within a short period of time and make it possible to study the role of adult hippocampal neurogenesis in a gain-of-function manner. More importantly, we provided evidences that this new DMS paradigm efficiently rescues behavioral phenotypes and gene expression profiles in learned helplessness mouse model, as well as restoring hippocampal LTP impaired by restraint stress in rats. These results support the notion that DMS is an efficient therapeutic treatment for major depression disorders and strongly suggest that the antidepressant effect of DMS on depression disorders may rely on the improvement of hippocampal adult neurogenesis and the correction of hippocampal synaptic plasticity.

## Methods

### Animals

Animals were group-housed with free access to water and food in the established animal houses, with a 12 hours light/dark cycle and a thermo-regulated environment. The use and care of animals complied with the guideline of the Biomedical Research Ethics Committee at the Shanghai Institutes for Biological Science, CAS. Adult (6–7 weeks old) male C57BL/6 mice (SLAC Laboratory Animal) were used in all experiments except electrophysiology trials which were performed on male Sprague Dawley rats (Animal House center, Kunming General Hospital, Kunming), weighing 220–250 g. *mecp2*^*308/y*^ mice (005439) and nestin-cre mice (003771) were purchased from Jackson lab; floxed *mecp2* (011918) were purchased from MMRRC at UC Davis.

### DMS treatment

For DMS treatment, mice with their cage were placed in DMS machine. Metal parts of cages were removed prior to DMS treatment. The applied program was described in Supplementary Figures. Briefly, twenty minutes successive trains of DMS were administered daily for different consecutive days depending on the purpose of experiments.14 days DMS were administered on retrovirus injected mice and 4 or 7 days DMS on Brdu labeled mice while 5 days DMS on acute induced depression mice. The control group conditions were identical to their DMS group but received sham stimulation. For electrophysiology experiments, control group received no treatment, Restraint stress (RS) group were restrained in the fixing cage 20 min each day for 7 days, RS + DMS group received 20 min DMS treatment in the fixing cage for 7 days. Electrophysiology studies were carried out 0.5 h later after the last restraint stress or DMS treatment.

### *In vivo* electrophysiology

Experiments were carried out on rats anesthetized with pentobarbital (60 mg/kg, i.p.), and core temperature was maintained at 37 ± 0.5°C. Recordings of field excitatory postsynaptic potentials (EPSPs) were made from the CA1 stratum radiatum of the hippocampus in response to ipsilateral stimulation of the Schaffer collateral/commissural pathway using techniques described previously [28,47]. Recording and stimulating electrodes were made by gluing together a pair of twisted Teflon-coated 90% platinum and 10% iridium wires (50 μm inner diameter, 75 μm outer diameter; World Precision Instruments, Sarasota, FL). The recording electrode was inserted 3.8 mm posterior to bregma and 2.8 mm right of the midline, and the stimulating electrode was inserted 4.8 mm posterior to bregma and 3.8 mm right of the midline. The optimal depth of the wire electrodes in the stratum radiatum of the CA1 area of the dorsal hippocampus was determined by electrophysiological criteria. Test EPSPs were evoked at a frequency of 0.033 Hz and at a stimulus intensity adjusted to give an EPSP amplitude 50% maximum response. The high frequency stimulation (HFS) protocol for inducing LTP consisted of 10 stimulus trains of 20 pulses at 200 Hz, with 2 s intertrain intervals. EPSP amplitude was expressed as mean ± S.E.M% of the baseline EPSP amplitude recorded over a 40-min baseline period, and amplitudes in the last 10-min of recording were averaged in one animal and then across animals to give a value for the group.

### Preparation and stereotaxic injection of retrovirus

We thank Drs. Hongjun Song (Johns Hopkins University School of Medicine) and Zhengang Yang (Institutes of Brain Science, Fudan University) for providing retroviral constructs and supernatant containing retrovirus. Cell debris were removed from supernatant through 0.22 μm filter and the filtered supernatant was centrifuged at 65000 g at 4°Cfor 2 h. Then supernatant was removed and 50 μl PBS was used to re-suspend virus. We seed 293 T to determine the titer of virus. High titer virus of 10^8^vg/ml was necessary to label new-born neurons in the adult DG.

For stereotaxic injection, it is critical to locate the exact hippocampus neurogenic area. The mouse was mounted onto the stereotaxic frame, orienting the head straight in terms of anterior-posterior axis and horizontally. Then we shaved a small area by a trimmer, cut the skin over the scull, cleaned up the blood in the wound.

The needle was firstly moved to the bregma and then moved posteriorly 2.0 mm and laterally 1.5 mm to the injection position. A small hole was made on the scull using an electric drill and the needle tip was moved down by 2.25 mm from the water level of the hole. 1 μl retrovirus solution was injected to one side.

For both Brdu and retrovirus stereotaxic injection statistics, each group (control, program1 and program5) has 4–5 mice. Each mouse was collected 9–12 slices from dorsal to ventral region of DG. And 1 or 2 intact neurons were chosen from each slice for analysis. ImageJ software (http://rsbweb.nih.gov/ij/) was used to trace the dendrites of new-born neuron. Then the dendritic length and tips number were auto-analyzed.

### Bromodeoxyuridine injection

Mice received four or seven days DMS treatment and Bronodeoxyuridine (Brdu) (50 mg/kg in saline, every two hours for four times, Sigma, flu/Ald, B5002) was intraperitoneally (i.p.) injected into the control or DMS group mice 12 hours after the last DMS treatment. Mice were killed two hours later after the last i.p. injection.

### Tissue preparation, immunostaining and imaging

Mice were transcardially perfused with 0.1 M cold PBS followed by 4% paraformaldehyde (PFA) fixation. The brains were post-fixed for 12 hours in 4% PFA and dehydrated in 30% sugar solution for another 12 hours. Coronal sections of 40-μm thickness were cut on the freezing microtome (LEICA CM1950) and stored in PBS.

For Brdu immunostaining, free-floating sections were incubated in 2 N HCl for 15 min at 37 degree then neutralized in 0.1 M boric acid solution (pH 8.5) for 10 min and washed by PBS for 3 times (each time for 5 minutes). Sections were incubated in mouse anti-Brdu antibody (1:1500; MAB3510) at 4 degree overnight and washed again then incubated in CF555 conjugated donkey anti-mouse secondary antibody (1:500; Biotum,20037) for 2.5 hours at room temperature.

For GFP immunostaining, free-floating sections were incubated in mouse anti-GFP antibody (1:1000;Santa Cruz, 9996) at 4 degree overnight and washed again then incubated in CF488 conjugated goat anti-mouse secondary antibody (1:500;Biotum,20010) for 2.5 hours (dark) at room temperature.

Immunostaining sections were photographed in Z-series stacks using a Nikon A1 confocal microscope. NIH Image J with NeuronJ plugin was used to count the Brdu-immunoreactive cell number in the dentate gyrus and to quantify the area of the dentate gyrus, also to analyze total dendritic length of GFP^+^ new-born neurons.

### Statistical analysis

The results were expressed as Mean ± S.E.M. Statistical significance (*P* < 0.05) was assessed using the two-tailed Student’s *t*-test.

### Learned helplessness paradigm

Mice were placed into the inescapable shock chamber. 360 scrambled foot shocks (0.6 mA) with varying duration (1-3s) and interval-episodes (1-15s) were delivered over two consecutive days. Control group did not receive foot shocks but were placed to the shock chamber with equal time. On the first and second days, 8 hours after foot shocks, mice were administered DMS. Then mice were administered DMS for another 3 days.

### Forced swim test

Mice were placed individually in the testing cylinder (33 cm high × 10 cm in diameter) containing water with 20 cm depth. The procedures were conducted as two minutes pretest followed by four minutes test and both sessions were videotaped. Observers scored the immobility (floating passively with slight movements) time in the test four minutes.

### Gamma irradiation

Mice were anesthetized with 0.7% Pentobarbital sodium at 10ul/g, and exposed to cranial irradiation at a field of 3.5×11mm above the hippocampus but other body parts were protected with a 75 mm lead shield. Caesium-137 was used as radioactive source operated by MDS Nordion GC-3000Elan. The dose rate was approximately 4 Gy per min. The procedure lasted 5 min and 30 sec, delivering a total of 20 Gy in the center.

### Light–dark transition

*mecp2*^*308/y*^ mutant mice were carried out light–dark transition test after daily DMS stimulation for 5 month at the age of 5–6 month. The chamber is divided into two unequal compartments by an opaque shelter with a hole (of height 4 cm) at the floor level. The smaller compartment (18cm×27cm×30cm) is painted black and covered by an opaque lid while the larger compartment (27cm×27cm×30cm) is uncovered and is illuminated by ceiling room lights. Firstly, mice were gently placed into the dark compartment. Transition between the two compartments and time spent in the light compartment were automatically recorded by the Ethovision videotracking system in a 10 min session.

### Quantitative real time PCR

Total RNA was collected from fresh hippocampus tissue for reverse transcription (Bio-Rad, 1708891). 2 × SYBR Green Master Mix (TOYOBO, QPK201) and QIAGEN Rotor-Gene Q machine were used in real time PCR experiments. Mouse MKP-1 real time primer: forward: CGCTTCTCGGAAGGATATGCT; reverse: GTCAATAGCCTCGTTGAACCAG. Fgf1b primers were used as reported [23].

## Competing interests

The authors declare that they have no competing interests.

## Authors’ contributions

LX and ZQ conceived and supervised the project. YZ performed the majority of the work. RRM and MT performed electrophysiology work on rat. ZFC, MH, ZRG, XL, DLD, participated in early stage of this work including immunohistochemistry and daily DMS treatment for animals. JW designed the DMS machine. XX and WHZ and CYL participated in testing and data collection of DMS machine on animals. ZQ wrote the manuscript, with contributions from other authors. All authors read and approved the final manuscript.

## Supplementary Material

Additional file 1: Figure S1 Illustration of DMS equipment and magnetic fields. (A) The positions of animal and DMS machine are showed. (B-C) Illustration of magnetic vector potential generated during Uniform and Linear phase by DMS equipment. (D-E) Illustration of Uniform and Linear magnetic fields generated by this equipment. Click here for file

Additional file 2: Figure S2 The effect of DMS to adult hippocampal neurogenesis. A) Hippocampal DG Ki67 and BrdU immunostaining on mice with continuous DMS with P5 treatment for 7 days. Blue: DAPI; Green: Ki67; Red BrdU staining. (B) Quantification of (A). (C) Hippocampal DG Ki67 immunostaining on mice with continuous DMS with P5 treatment for 7 days. Blue: DAPI; Green: Ki67 staining. Lower right panel: Amplification of boxed area of (A) panel. (E) Hippocampal DG GFAP and BrdU immunostaining on mice with continuous DMS with P5 treatment for 7 days. Blue: DAPI; Green: GFAP; Red BrdU staining. (F) Quantification of (E). (G) Hippocampal DG GFAP immunostaining on mice with continuous DMS with P5 treatment for 7 days. Blue: DAPI; Green: GFAP staining. Lower right panel: Amplification of boxed area of (G) panel. Scale bar = 100 μm. Click here for file

Additional file 3: Figure S3 The effect of DMS to adult hippocampal neurogenesis (Continued). (A) Hippocampal DG Tbr2 and BrdU immunostaining on mice with continuous DMS with P5 treatment for 7 days. Blue: DAPI; Green: Tbr2; Red BrdU staining. (B) Quantification of (A). (C) Hippocampal DG Tbr2 immunostaining on mice with continuous DMS with P5 treatment for 7 days. Blue: DAPI; Green: Ki67 staining. Lower right panel: Amplification of boxed area of (C) panel. (E) Hippocampal DG DCX and BrdU immunostaining on mice with continuous DMS with P5 treatment for 7 days. Blue: DAPI; Green: DCX; Red BrdU staining. (F) Quantification of (E). (G) Hippocampal DG DCX immunostaining on mice with continuous DMS with P5 treatment for 7 days. Blue: DAPI; Green: DCX staining. Lower right panel: Amplification of boxed area of (G) panel. Scale bar = 100 μm. Click here for file

Additional file 4: Figure S4 The effect of DMS to development of mature hippocampal DG neurons. Example pictures of Golgi staining for hippocampal DG neuron in control (A) and DMS (B) condition. (C) Golgi staining for spines on hippocampal DG neurons. (D) Quantification of (C). (E) Immunohistochemistry for c-fos protein in laternal habenula region after DMS with P5 treatment for 4 days. (F) Quantification of (E). Click here for file

Additional file 5: Figure S5 The effect of DMS on stress-induced depressive phenotypes in rodent models. (A) Outline of foot-shock-induced depressive behavioral paradigm and DMS treatment experiments. (B-E) Behavioral effects of learned helplessness mouse model with DMS treatment during various time points. Values represent mean (±) SEM (Animal amount: control n = 15, Footshock n = 19, Footshock with DMS n = 15; **: P < 0.005, two-tailed student’s t-test. Click here for file

Additional file 6: Figure S6 IR inhibits adult hippocampal neurogenesis in rodents. (A) Outline of gamma irradiation (IR) and BrdU injection experimentsHippocampal DG BrdU immunostaining on control mice (B), and with IR treatment (C). Red: BrdU; Blue: DAPI staining. (D) Quantification of (C,D). Values represent mean (±) SEM (n = 12-16 for each condition; *: P < 0.05, two-tailed student’s t-test). Scale bar = 100 μm. Click here for file
