# Supplementary figures and images for: Deep-brain magnetic stimulation promotes adult hippocampal neurogenesis and alleviates stress-related behaviors in mouse models for neuropsychiatric disorders
Source: Mol Brain. 2014 Feb 11;7:11. doi: 10.1186/1756-6606-7-11 (PMC3928113; doi:10.1186/1756-6606-7-11)

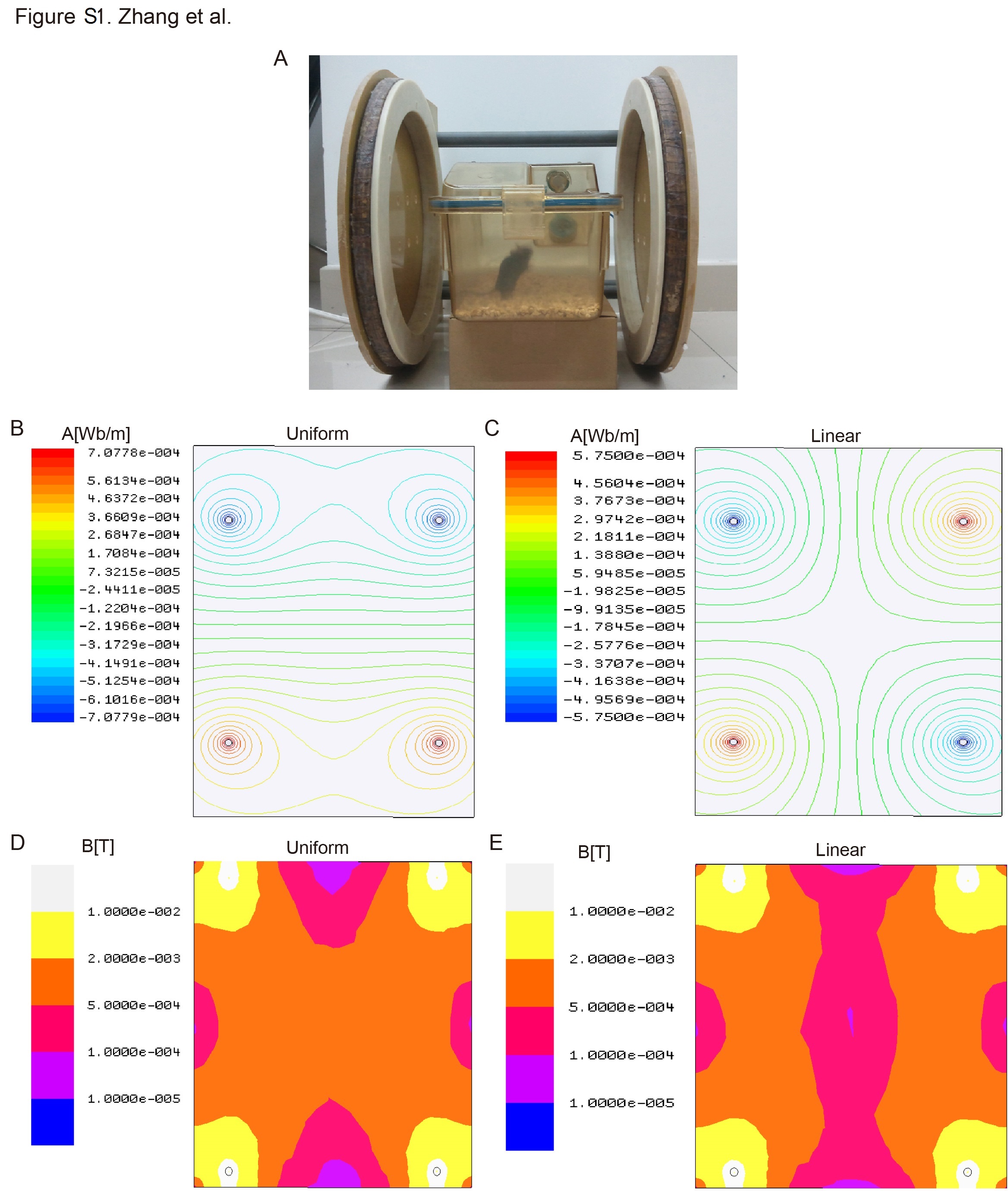

Supplement: Additional file 1: Figure S1 — Illustration of DMS equipment and magnetic fields. (A) The positions of animal and DMS machine are showed. (B-C) Illustration of magnetic vector potential generated during Uniform and Linear phase by DMS equipment. (D-E) Illustration of Uniform and Linear magnetic fields generated by this equipment. [file 1756-6606-7-11-S1.jpeg]

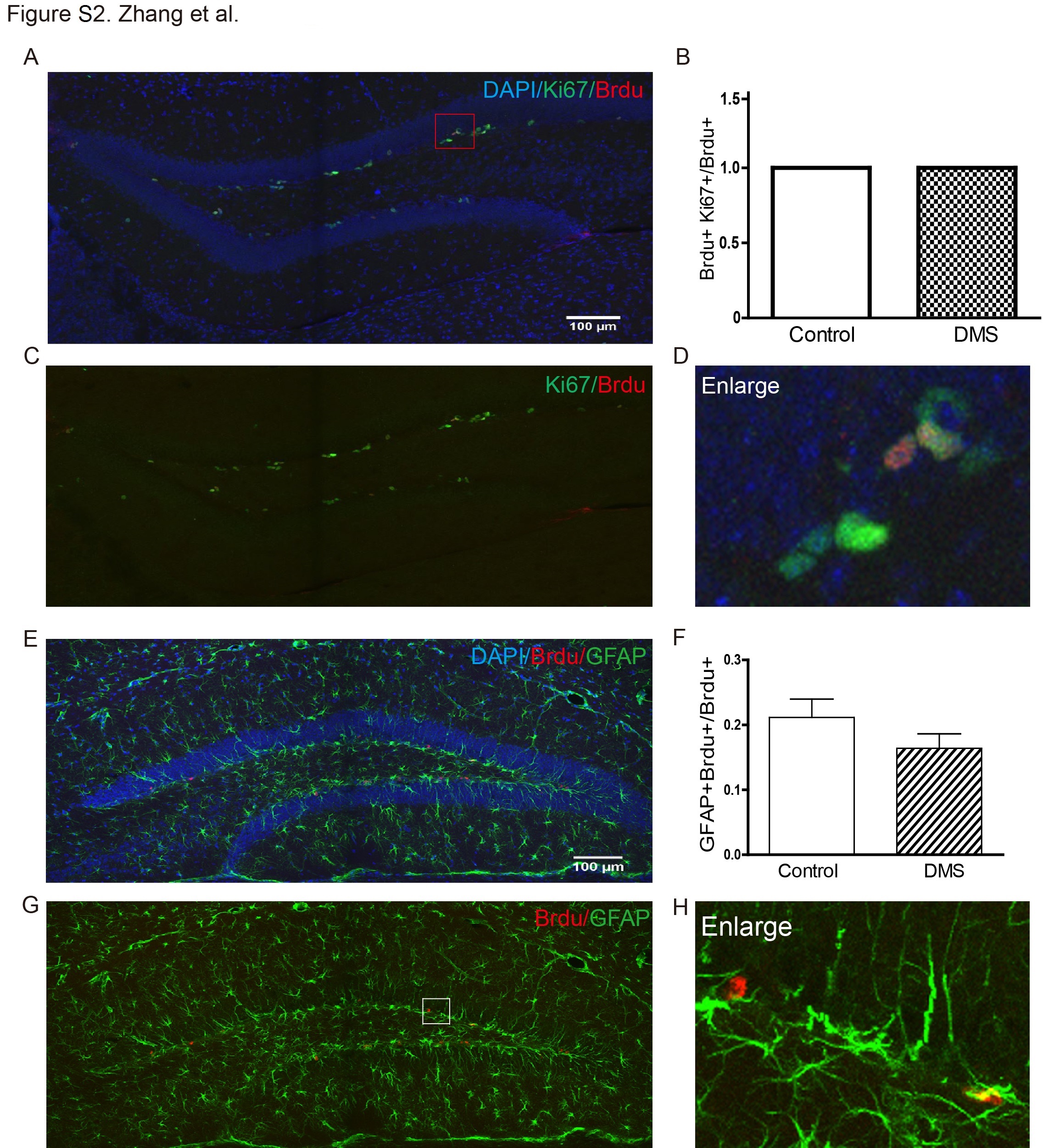

Supplement: Additional file 2: Figure S2 — The effect of DMS to adult hippocampal neurogenesis. A) Hippocampal DG Ki67 and BrdU immunostaining on mice with continuous DMS with P5 treatment for 7 days. Blue: DAPI; Green: Ki67; Red BrdU staining. (B) Quantification of (A). (C) Hippocampal DG Ki67 immunostaining on mice with continuous DMS with P5 treatment for 7 days. Blue: DAPI; Green: Ki67 staining. Lower right panel: Amplification of boxed area of (A) panel. (E) Hippocampal DG GFAP and BrdU immunostaining on mice with continuous DMS with P5 treatment for 7 days. Blue: DAPI; Green: GFAP; Red BrdU staining. (F) Quantification of (E). (G) Hippocampal DG GFAP immunostaining on mice with continuous DMS with P5 treatment for 7 days. Blue: DAPI; Green: GFAP staining. Lower right panel: Amplification of boxed area of (G) panel. Scale bar = 100 μm. [file 1756-6606-7-11-S2.jpeg]

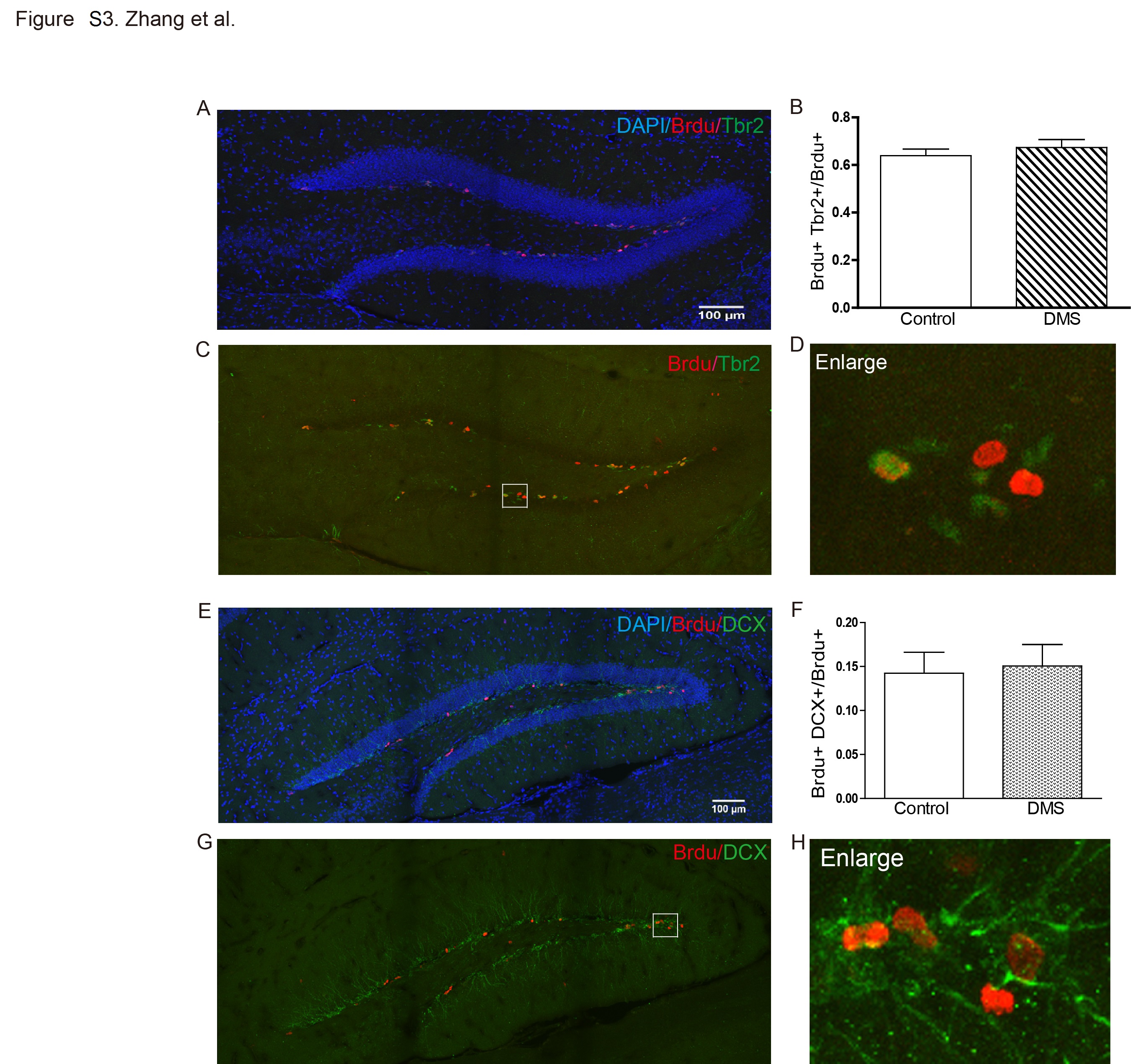

Supplement: Additional file 3: Figure S3 — The effect of DMS to adult hippocampal neurogenesis (Continued). (A) Hippocampal DG Tbr2 and BrdU immunostaining on mice with continuous DMS with P5 treatment for 7 days. Blue: DAPI; Green: Tbr2; Red BrdU staining. (B) Quantification of (A). (C) Hippocampal DG Tbr2 immunostaining on mice with continuous DMS with P5 treatment for 7 days. Blue: DAPI; Green: Ki67 staining. Lower right panel: Amplification of boxed area of (C) panel. (E) Hippocampal DG DCX and BrdU immunostaining on mice with continuous DMS with P5 treatment for 7 days. Blue: DAPI; Green: DCX; Red BrdU staining. (F) Quantification of (E). (G) Hippocampal DG DCX immunostaining on mice with continuous DMS with P5 treatment for 7 days. Blue: DAPI; Green: DCX staining. Lower right panel: Amplification of boxed area of (G) panel. Scale bar = 100 μm. [file 1756-6606-7-11-S3.jpeg]

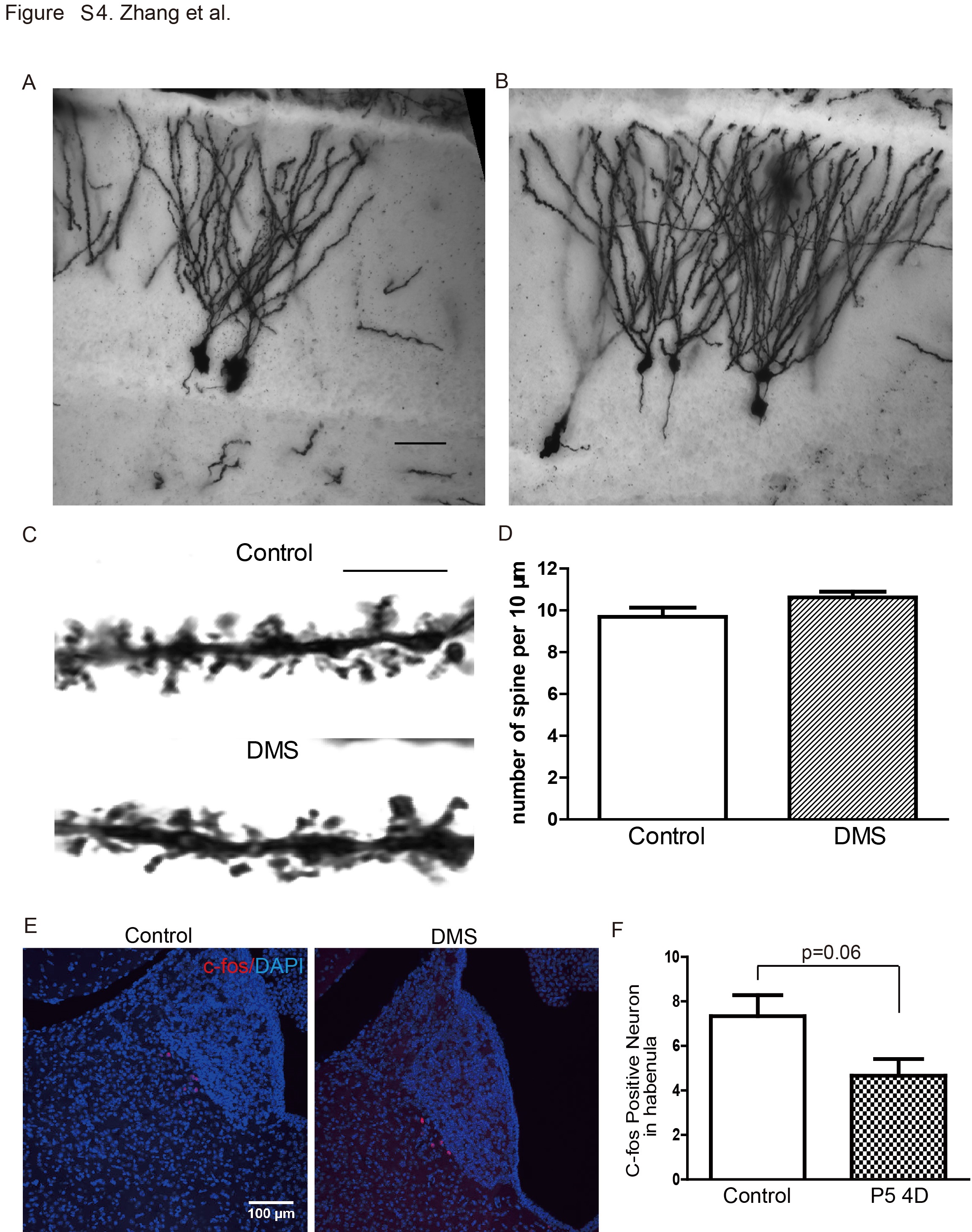

Supplement: Additional file 4: Figure S4 — The effect of DMS to development of mature hippocampal DG neurons. Example pictures of Golgi staining for hippocampal DG neuron in control (A) and DMS (B) condition. (C) Golgi staining for spines on hippocampal DG neurons. (D) Quantification of (C). (E) Immunohistochemistry for c-fos protein in laternal habenula region after DMS with P5 treatment for 4 days. (F) Quantification of (E). [file 1756-6606-7-11-S4.jpeg]

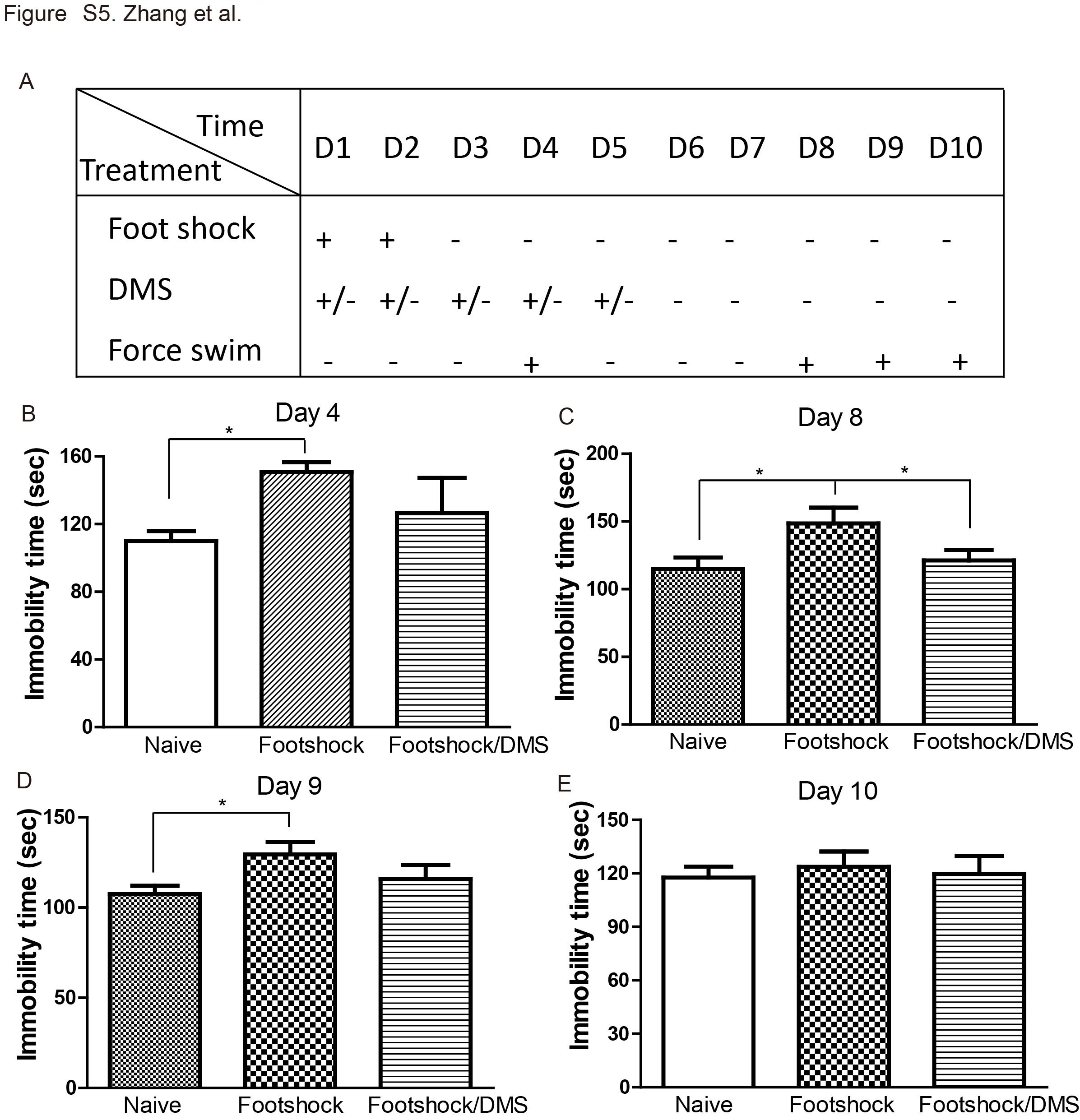

Supplement: Additional file 5: Figure S5 — The effect of DMS on stress-induced depressive phenotypes in rodent models. (A) Outline of foot-shock-induced depressive behavioral paradigm and DMS treatment experiments. (B-E) Behavioral effects of learned helplessness mouse model with DMS treatment during various time points. Values represent mean (±) SEM (Animal amount: control n = 15, Footshock n = 19, Footshock with DMS n = 15; **: P < 0.005, two-tailed student’s t-test. [file 1756-6606-7-11-S5.jpeg]

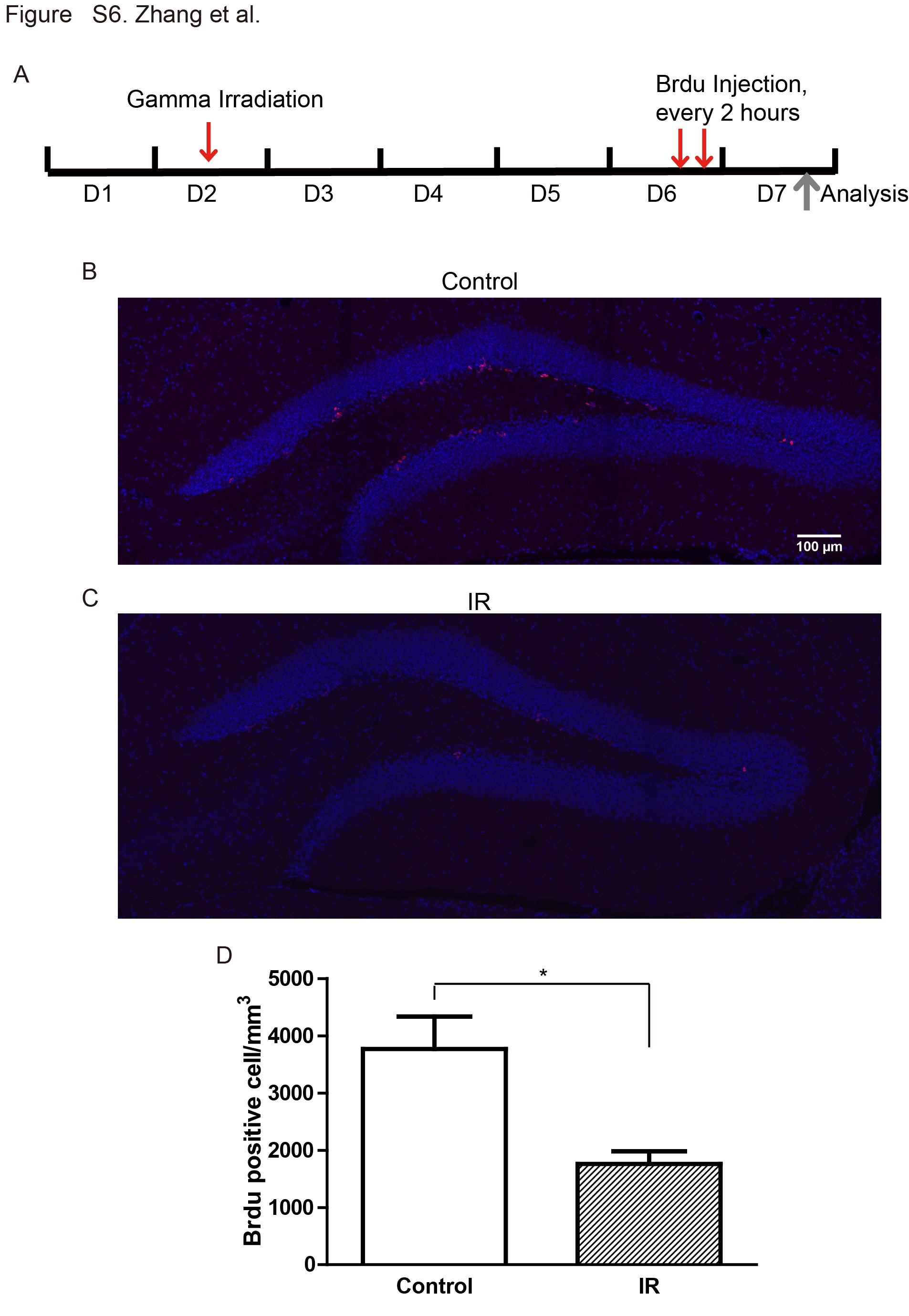

Supplement: Additional file 6: Figure S6 — IR inhibits adult hippocampal neurogenesis in rodents. (A) Outline of gamma irradiation (IR) and BrdU injection experimentsHippocampal DG BrdU immunostaining on control mice (B), and with IR treatment (C). Red: BrdU; Blue: DAPI staining. (D) Quantification of (C,D). Values represent mean (±) SEM (n = 12-16 for each condition; *: P < 0.05, two-tailed student’s t-test). Scale bar = 100 μm. [file 1756-6606-7-11-S6.jpeg]
